# The acute effects of walking exercise intensity on systemic cytokines and oxidative stress

**DOI:** 10.1007/s00421-018-3930-z

**Published:** 2018-07-14

**Authors:** Malcolm Brown, Conor M. McClean, Gareth W. Davison, John C. W. Brown, Marie H. Murphy

**Affiliations:** 1 Sport and Exercise Sciences Research Institute, Ulster University, Jordanstown, County Antrim BT37 0QB Northern Ireland, UK; 2 School of Nursing and Midwifery, Medical Biology Centre, Queen’s University Belfast, Belfast, BT9 7BL Northern Ireland, UK

**Keywords:** Walking, High intensity intermittent exercise, Oxidative stress, Cytokine

## Abstract

**Purpose:**

Oxidative stress is associated with tissue cytokine secretion although the precise mechanism(s) underpinning this relationship during high intensity intermittent exercise remains unclear. This study investigates the acute response to a bout of high intensity intermittent walking (HIIW), compared to continuous moderate intensity walking (CMW), on various cytokines and biomarkers of oxidative stress.

**Methods:**

Seventeen (*n* = 17) apparently healthy male participants (aged 22.6 ± 4.6 years; $\dot{V}{\text{O}}_{2} \max$: 53.7 ± 7.1 ml kg^−1^ min^−1^) undertook a randomised crossover study consisting of two exercise trials: (1) HIIW requiring 3 × 5 min bursts at 80% $\dot{V}{\text{O}}_{2} \max$ (each separated by 5 min of walking at 30% $\dot{V}{\text{O}}_{2} \max$) and (2) CMW (60% $\dot{V}{\text{O}}_{2} \max$ for 30 min). Each trial was separated by 7 days. Venous blood samples were obtained pre-exercise, post-exercise and at 2, 4, 24 and 48 h post-exercise for determination of systemic inflammation (IL-6 and TNF-α), lipid soluble antioxidants and oxidative stress (LOOH, H_2_O_2_ and the ascorbyl free radical).

**Results:**

Both IL-6 and TNF-α increased immediately post exercise, regardless of intensity and remained elevated until at least 4 h (main effect for time; *p* < 0.05). While there was no change in either lipid peroxidation or free radical metabolism (Asc· and H_2_O_2_), α-tocopherol increased (pooled HIIW and CMW, *p* < 0.05), whereas lycopene decreased at 2 h post HIIW (*p* < 0.05).

**Conclusion:**

Bouts of both HIIW and CMW promote cytokine secretion post exercise, and this seems to be independent of oxidative stress. Further investigation is required to assess how such changes may underpin some of the transient health benefits of exercise.

## Introduction

Recent research postulates that balanced redox status and chronic systemic low-grade inflammation are potent mediators of homeostasis (Gleeson et al. 2011). Exercise has the capacity to transiently provoke a response in both biological systems and as such, has received increased research attention (Steinberg et al. 2007). It is now well established that enhanced cellular respiration during exercise leads to the generation of partially reduced oxygen derivatives, termed reactive oxygen species (ROS) (Davison et al. 2012). ROS react indiscriminately causing damage to molecular components and may serve as key proponents in several diseases (Sies 2015). Normally antioxidant mechanisms effectively reduce their potency although if abundantly created, coping capacities become overwhelmed and a state of oxidative stress ensues (Powers and Jackson 2008).

While excessive amounts of ROS are detrimental, transient exercise-induced changes are now recognised as integral agents in promoting adaptation (Radak et al. 2013). Cellular stress (i.e. disruption to cellular homeostasis and not just oxidative stress) may serve as a key stimulant of ‘*myokine*’ expression and the downstream anti-inflammatory cascade (Scheele et al. 2009). Direct empirical evidence of this supposed action is limited, and somewhat controversial, yet several reviews support this plausible mechanism (Scheele et al. 2009; Welc and Clanton 2013; Peake et al. 2015; Ost et al. 2016). The underlying molecular processes remain equally unexplored, but may arise from oxidative or endoplasmic reticulum stress, subsequent induction of p38 mitogen-activated protein kinase (MAPK) and putative transcriptional regulation of NF-κB (nuclear factor kappa B) (Scheele et al. 2009; Powers et al. 2011; Welc and Clanton 2013, Ost et al. 2016). Several reports advocate that p38 MAPK is activated in response to the physiological perturbations associated with acute high-intensity exercise, and the corresponding increases in muscular ROS (Kefaloyianni et al. 2006; Kramer and Goodyear 2007). Likewise, NF-κB is phosphorylated by ROS, promoting nuclear translocation and transcription of various cytokines and chemokines (Powers et al. 2011; Chu 2013; He et al. 2016). Thus muscular cytokine kinetics appear redox sensitive and given both share overlapping signalling pathways, conceivably ROS directly influence a parallel rise in cytokine secretion (Sallam and Laher 2016). Although at this time, little is known of the impact of exercise intensity and pattern on the interactive oxidative and inflammatory mechanisms, especially in the post-exercise period.

A rise in IL-6 secretion has been consistently reported in venous circulation following exercise, proportionally related to exercise intensity, duration and muscle mass recruited (Petersen and Pedersen 2005). Normally prolonged or unaccustomed bouts of exercise report the greatest increase, although a modest rise has been detected with moderate intensity walking (Nieman et al. 2005; Mendham et al. 2011). IL-6 may mediate the immunosuppressive cascade and influence metabolic activity, however the transient effects of a single bout of HIIW remain unclear, even though several cardioprotective effects have been identified using similar strategies (Hood et al. 2011; Little et al. 2011a).

Intense, interval based exercise has recently emerged as a time efficient strategy to elicit a range of health and fitness benefits including improved metabolism and immunity, oxidative capacity and antioxidant defence (Little et al. 2011b; Gibala et al. 2012). Previously published studies examining the impact of intense, interval exercise often deploy Wingate-like testing procedures using cycle ergometers, which may be poorly tolerated (Boutcher 2011). There is now a need for more practical strategies and in recent years effective models (i.e. decreasing absolute intensity, increasing duration and reducing resting intervals) have been presented (Leggate et al. 2010; Gibala et al. 2012). In fact, walking satisfies all three criterion and exercise intensity can be readily manipulated by increasing and decreasing cadence (Murphy et al. 2007). The threshold range for high intensity intermittent exercise has since been reduced, previously from near maximal bursts, to bouts beyond 75% $\dot{V}{\text{O}}_{2} \max$ now deemed sufficient to induce a host of health benefits (Francois and Little 2015). Currently, there is a paucity of evidence characterising the transient response of cytokines and oxidative stress following differing walking intensities, particularly the influence of HIIW on these key biochemical parameters. Thus the aim of this study was to measure the differing biochemical response to single bouts of intermittent and continuous walking.

## Methodology

### Participant characteristics

Following ethical approval from the University Ethics committee and in accordance with the Declaration of Helsinki, seventeen (*n* = 17) apparently healthy and recreationally active (approximately 2-h week^−1^ exercise) male participants (22.6 ± 4.6 years; 179.2 ± 5.6 cm; 79 ± 10.6 kg; $\dot{V}{\text{O}}_{2} \max$: 53.7 ± 7.1 ml kg^−1^ min^−1^) were recruited. Prior to commencing the study, all participants completed a health history questionnaire to ensure suitability and provided informed consent after full study details were disclosed. All participants were non-smokers and free from medication and antioxidant supplementation.

### Experimental design

Participants completed a randomised (number generated) crossover study consisting of two trials: (1) intermittent vigorous walking exercise (HIIW) and (2) continuous moderate walking exercise (CMW). Each trial was separated by 7 days and participants were asked to refrain from exercise and alcohol consumption 24 h prior to each trial. Participants recorded their dietary intake 24 h prior to testing and were asked to replicate this for the second trial. Participants were tested following a standard 10 h overnight fast.

### Maximal oxygen consumption

Prior to experimental trials, participants undertook an incremental $\dot{V}{\text{O}}_{2} \max$ test on a motorised treadmill (H-P Cosmos, Germany) to exhaustion. Oxygen uptake was measured using a standard calibrated laboratory gas analysis system (Cosmed Quarkb^2^, Italy). Heart rate (Polar Electro, Finland) and perceived exertion was monitored continuously with a valid $\dot{V}{\text{O}}_{2} \max$ confirmed using the following criteria (1) the respiratory exchange ratio ≥ 1.15 (2) a clear plateau in mean oxygen uptake (< 2 ml kg^−1^ min^−1^) and (3) a heart rate within 10 beats·min^−1^ of age predicted maximum (208–0.7 × age) (Howley et al. 1995).

### Exercise protocol

Participants completed bouts of HIIW (3 × 5 min at 80% $\dot{V}{\text{O}}_{2} \max$ separated by 3 × 5 min at 30% $\dot{V}{\text{O}}_{2} \max$) and CMW (60% $\dot{V}{\text{O}}_{2} \max$ for 30 min) in a random order. The vigorous bout was devised in accordance with recent recommendations (Gibala et al. 2012; Francois and Little 2015) and finalised following a pilot trial. Participants were instructed to walk for the entire exercise duration. The speed and incline of the treadmill was adjusted to ensure each participant achieved and remained at the desired intensity.

### Biochemical analysis

#### Venous blood sampling

Venous blood samples were obtained at baseline, immediately post exercise, 2 and 4 h post exercise via a 22-gauge intravenous cannula (Biovalve Safe, Vygon, UK) inserted into a prominent antecubital fossa vein. Blood was drawn into serum clot activator and K_3_EDTA vacutainers (Greiner Bio-One, Austria). Following collection, serum tubes were allowed to clot at room temperature for approximately 10 min while K_3_EDTA tubes were placed on ice. Blood tubes were then centrifuged at 3500 rpm for 10 min at 4 °C (Hettich, Germany). Serum and plasma were extracted and stored at − 80 °C prior to biochemical analysis. Follow up sampling also occurred at 24 and 48 h post exercise using venepuncture.

### Measurement of cytokines (IL-6 & TNF-α)

IL-6 and TNF-α were determined in plasma by enzyme-linked immunosorbent assay (ELISA) according to the manufacturer instructions (BioLegend, CA, USA). Absorbance was read spectrophotometrically using a microplate reader (EL 808, BioTek Instruments, USA) at 450 nm in conjunction with Gen5 software. Concentration was calculated using curve-fitting software with a 4-parameter logistics algorithm (ReadFit Pro 2014, Hitachi Solutions).

### Measurement of endothelin-1

Endothelin-1 was determined pre and immediately post exercise in serum using a Quantikine® ELISA (R & D Systems, UK). The absorbance was measured at 450 nm using a microplate reader (EL808, BioTek Instruments, USA). Concentration was calculated using curve-fitting software with a 4-parameter logistics algorithm (ReadFit Pro 2014, Hitachi Solutions).

### Measurement of lipid hydroperoxides (LOOH)

Lipid hydroperoxides were measured in serum using the ferrous iron/xylenol orange (FOX) assay. The reagent was prepared using 25 mM L^−1^ of sulphuric acid (H_2_SO_4_), 250 µM L^−1^ of ammonium ferrous sulphate, 100 µM L^−1^ of sorbitol and 100 µM L^−1^ of xylenol orange in distilled water. 90 µL of serum was mixed with 900 µL of reagent, incubated at room temperature for 30 min in darkness and read at 560 nm using a spectrophotometer (UV mini-1240 Shimadzu, Mason Technologies, Ireland).

### Measurement of hydrogen peroxide (H_2_O_2_)

H_2_O_2_ was analysed in serum using an OxiSelect™ colorimetric assay according to the manufacturer instructions (Cell Biolabs Inc, CA, USA). The absorbance was immediately read at 560 nm by a microplate reader (FLUOstar Omega, BMG LABTEC, Germany).

### Electron paramagnetic resonance (EPR) spectroscopic analysis

The ascorbyl radical was measured in plasma using EPR spectroscopy. 1 mL of plasma was mixed thoroughly with 1 mL of dimethyl sulfoxide (DMSO) in a glass test tube. 1 mL of solution was then drawn into a sterile syringe and flushed into the EPR cavity. All samples were analysed at room temperature using a calibrated Bruker EMX series X-band EPR spectrometer (Bruker, Germany). The spectrometer parameter conditions were set as follows: frequency (9.785 GHz); microwave power (20 mW); modulation frequency (100 kHz) and modulation amplitude (1.194 G) for three sweeps. Spectral parameters were obtained using commercially available software (Bruker Win EPR System, Version 3.2) and filtered identically. The relative concentration of ascorbyl radical was determined by measuring signal intensity.

### Determination of lipid soluble antioxidants

Endogenous lipid soluble antioxidants were measured using the simultaneous high performance liquid chromatography (HPLC) assay of Thurnham et al. (1988). Plasma samples were measured under the same testing parameters outlined in McClean et al. (2015) for α-tocopherol, γ-tocopherol, retinol, lycopene, α-carotene and β-carotene at changing wavelengths of 292, 325 and 450 nm. Results were interpreted by Empower software (Version 2, Waters Corp, USA).

### Statistical analysis

Statistical analysis was performed using SPSS version 22 (IBM, Hampshire, UK). Data were analysed using a two-way repeated measures analysis of variance (ANOVA) with one between (trial) and one within (time) subject factor. For significant interaction effects, within subject factors were further analysed using a Bonferroni-corrected paired samples *t* test (*p* < 0.025). Between subject differences were analysed by one-way ANOVA with a posteriori Tukey Honestly Significant Difference (HSD) test. In the event of a main effect for time, data was pooled and paired sample *t* tests were performed. The alpha level was set at *p* < 0.05. A prospective power calculation, factoring critical difference, was conducted using the Altman method (Altman 1980). Retrospective power calculations were carried out while using SPSS. All data within are expressed as mean ± SEM (standard error of the mean) unless otherwise stated.

## Results

### Cytokine response

**Fig. 1:**
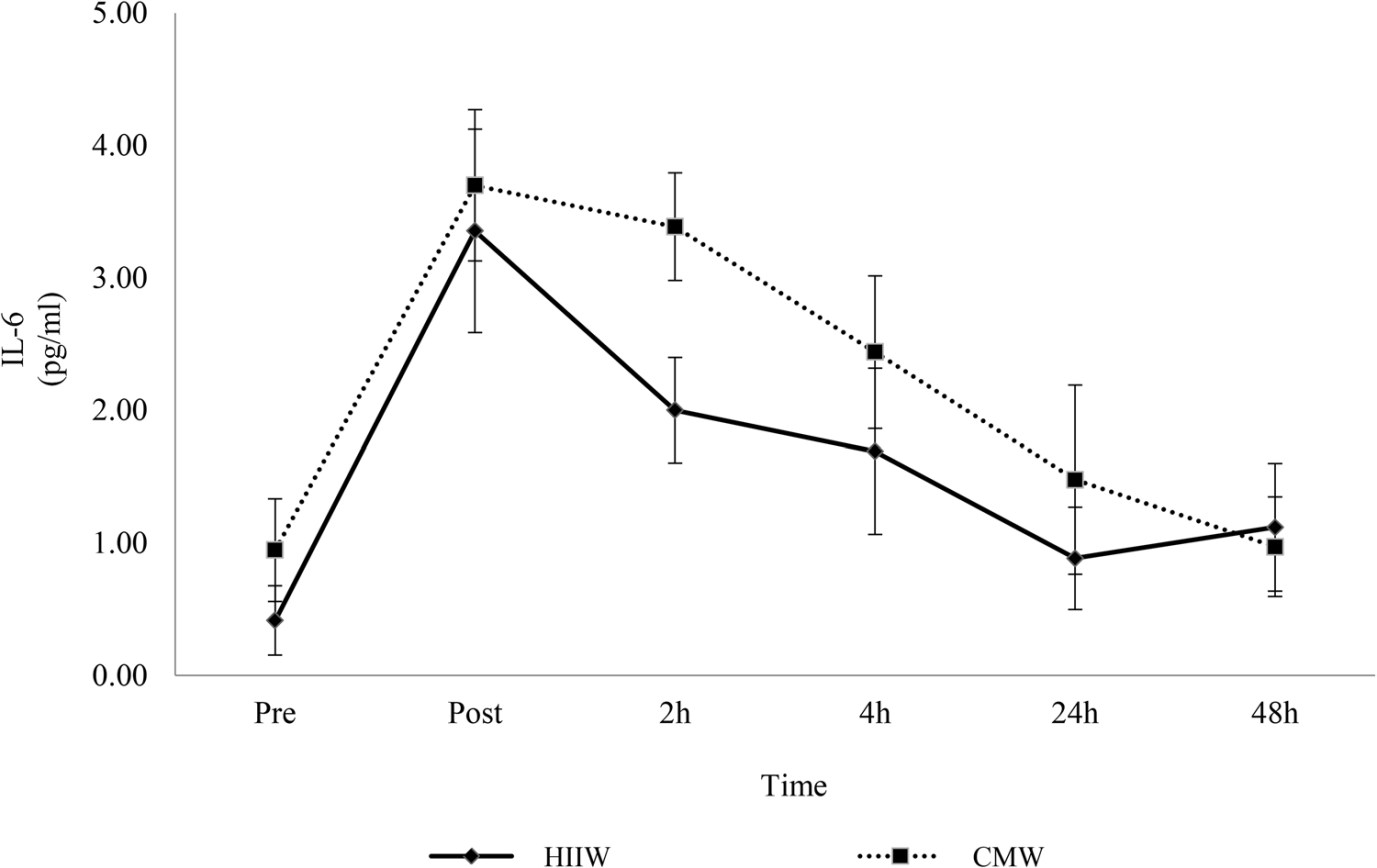
Effects of walking intensity on IL-6 over time (*n* = 17). Main effect for time at post exercise, 2 h and 4 h versus baseline (*p* < 0.05; pooled HIIW and CMW data)

**Fig. 2:**
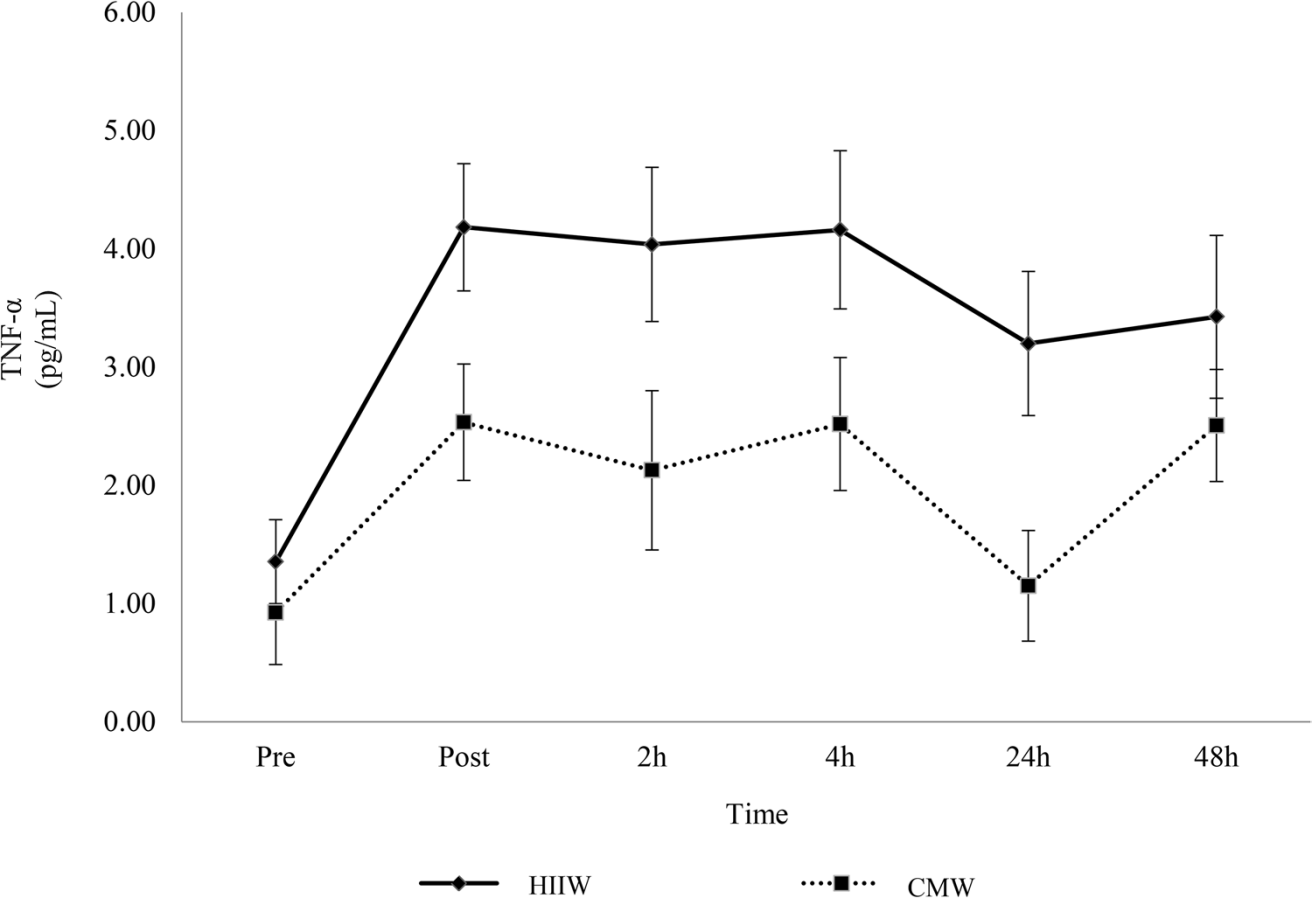
Effects of walking intensity on TNF-α over time (*n* 17). Main effect for time at post exercise, 2, 4, 24 and 48 h versus baseline (*p* < 0.05; pooled HIIW and CMW data)

Increases in IL-6 were observed following exercise in both conditions but no significant differences were observed between the trails (main effect for time, *p* < 0.05; time x group interaction, *p* > 0.05). Compared to pre-exercise, IL-6 increased at post exercise, 2 and 4 h (*p* < 0.05) before returning to baseline by 24 h (Fig. 1). Peak IL-6 occurred immediately post exercise. Likewise, no changes were observed between groups for TNF-α over time (*p* > 0.05). There was, however, a main effect for time (*p* < 0.05) as TNF-α increased from baseline throughout the post-exercise period (*p* < 0.05) (Fig. 2). TNF-α peaked immediately post exercise (mean: 3.36 pg/ml) with similar concentrations again at 4 h (mean: 3.34 pg/ml).

### Endothelin-1

**Fig. 3:**
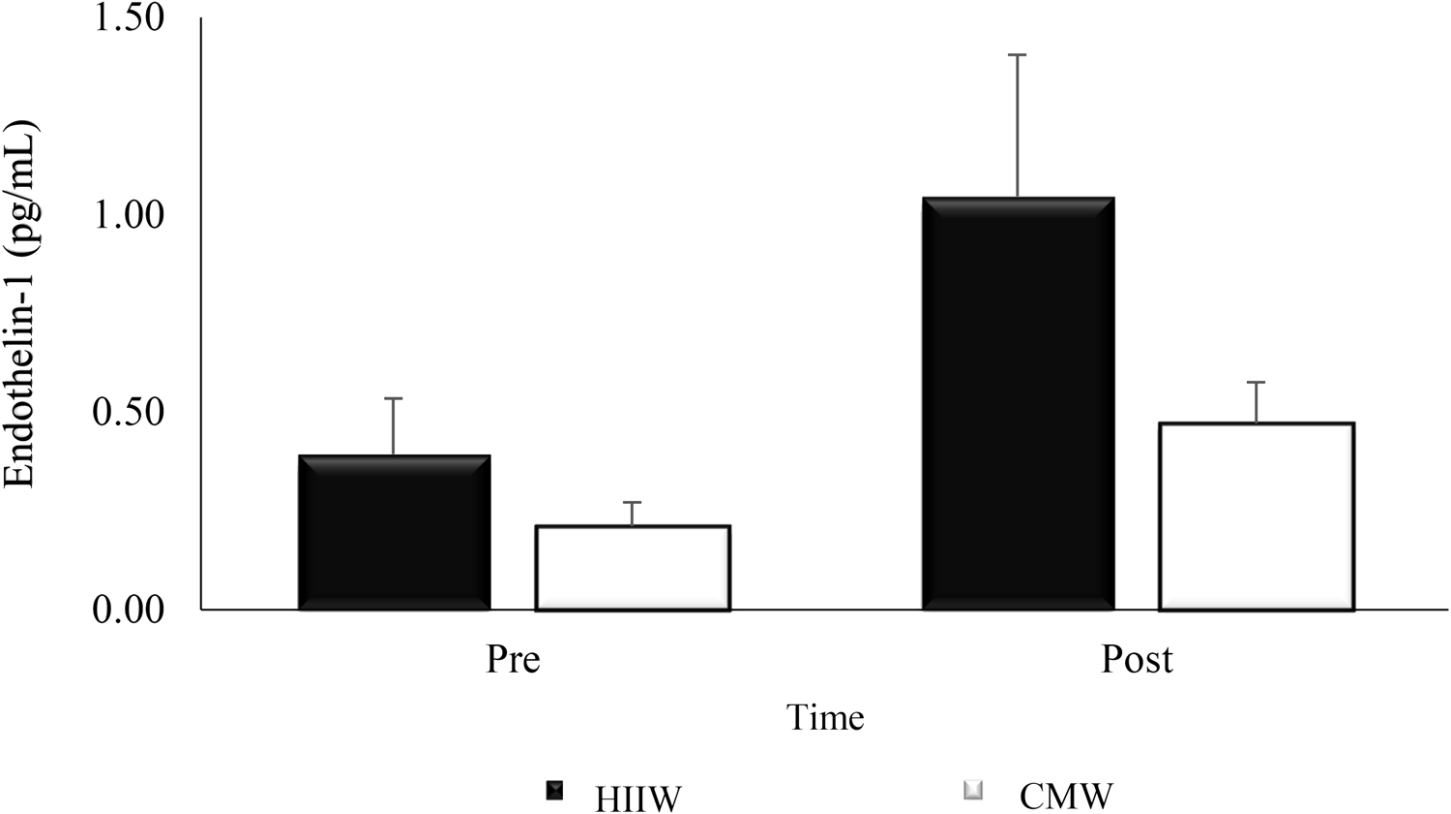
Effects of exercise intensity on endothelin-1 over time (*n* 17). Main effect for time at post exercise versus baseline (*p* < 0.05; pooled HIIW and CMW data)

There was no significant change from pre to post exercise between conditions (time × group interaction, *p* > 0.05). A main effect for time (*p* < 0.05) was detected as endothelin-1 increased post exercise in both trials (*p* < 0.05) (Fig. 3).

### Biomarkers of oxidative stress/damage

Table 1 shows no changes in any markers of oxidative stress (LOOH, H_2_O_2_ and ascorbyl radical) within or between conditions over time (*p* > 0.05).

**Table 1:** Biomarkers of oxidative stress following exercise over time (*n* = 17)

Biomarker	Trial	Pre	Post	2 h	4 h	24 h	48 h
LOOH (µΜ L^−1^)	HIIW	1.14 ± 0.05	1.19 ± 0.05	1.23 ± 0.05	1.22 ± 0.05	1.20 ± 0.05	1.19 ± 0.04
	CMW	1.22 ± 0.07	1.27 ± 0.05	1.23 ± 0.04	1.25 ± 0.06	1.20 ± 0.05	1.17 ± 0.06
H_2_O_2_ (µΜ/L)	HIIW	39.4 ± 3.61	41.4 ± 3.72	41.7 ± 3.64	44.9 ± 3.34	38.0 ± 3.95	39.5 ± 3.58
	CMW	39.4 ± 3.86	42.1 ± 3.56	43.6 ± 3.53	42.8 ± 3.33	38.9 ± 3.52	41.1 ± 2.98
Asc· (arbitrary units)	HIIW	340 ± 65	366 ± 61	426 ± 68	381 ± 77	428 ± 85	352 ± 78
	CMW	447 ± 80	447 ± 84	557 ± 78	473 ± 77	544 ± 70	439 ± 75

*CMW* continuous moderate, *HIIW* high intensity intermittent walking

### Lipid soluble antioxidants

**Fig. 4:**
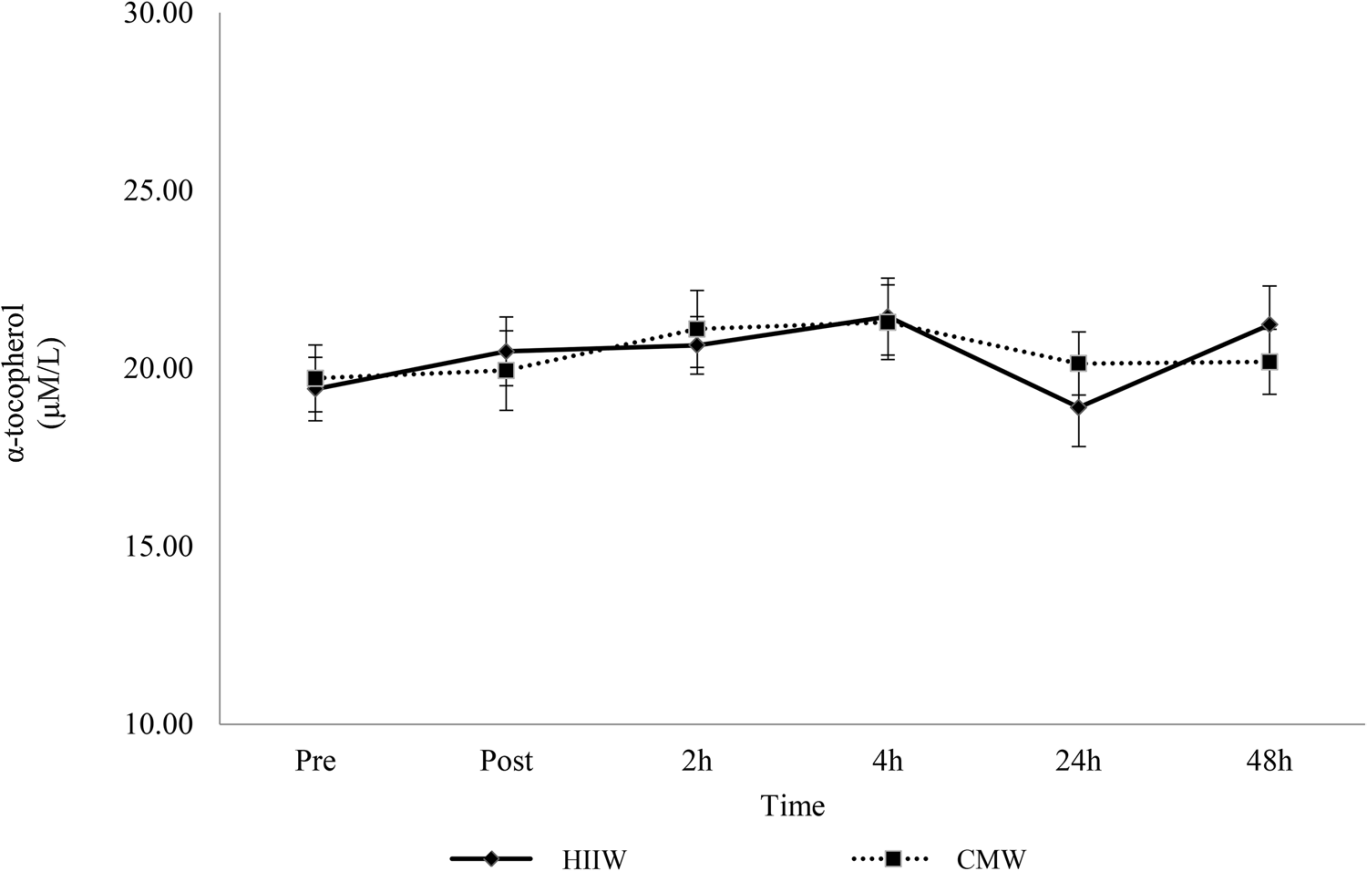
Effects of walking intensity on α-tocopherol over time (*n* 17). Main effect for time at 2 and 4 h versus baseline (*p* < 0.05; pooled HIIW and CMW data)

**Fig. 5:**
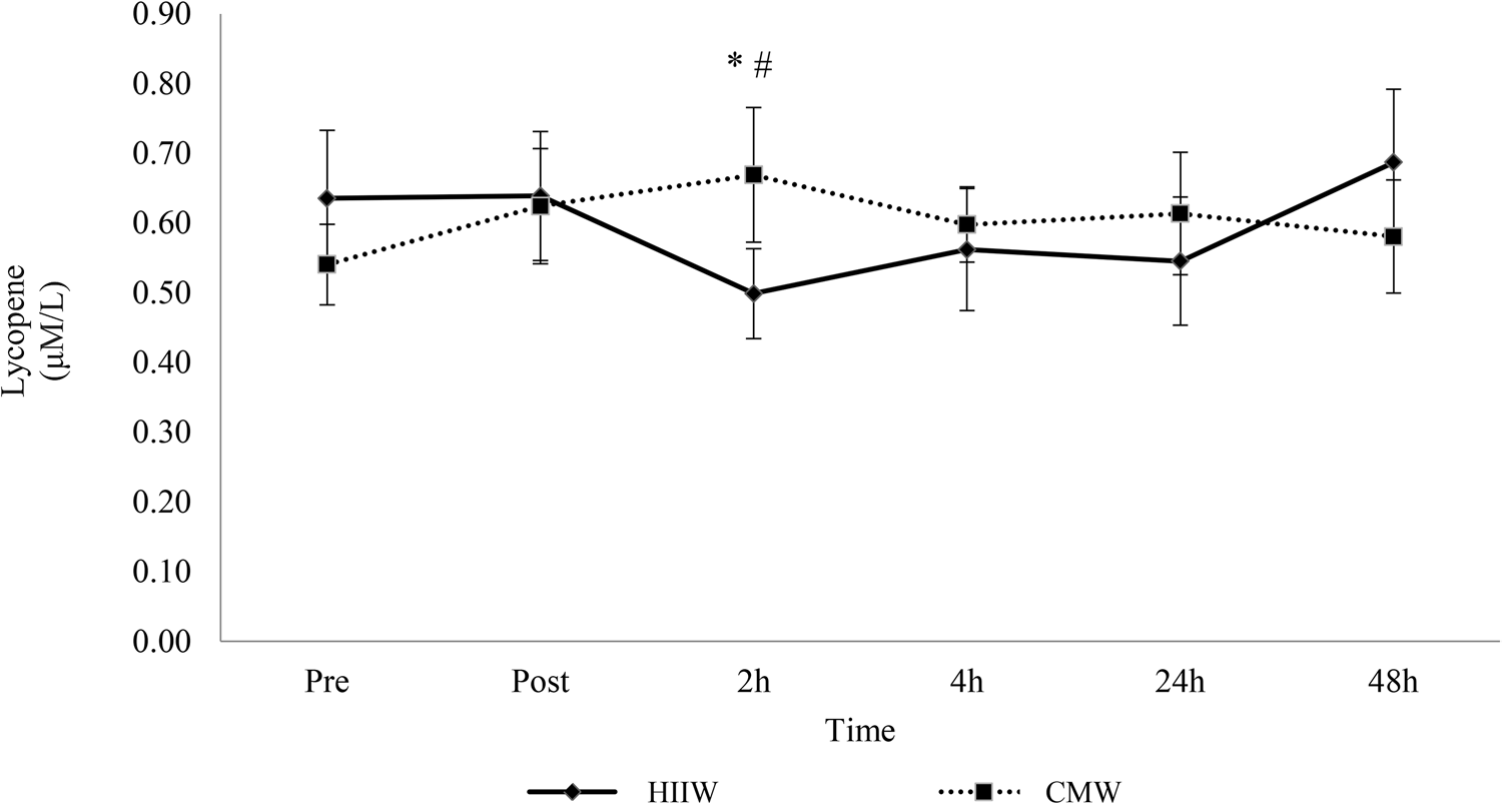
Effects of walking exercise intensity on lycopene over time (*n* 17). Lycopene decreased at 2 h versus baseline (*) and post-exercise (#) (*p* < 0.05) in the HIIW trial only

There was no change for selected lipid soluble antioxidants (retinol, γ-tocopherol, α-carotene and β-carotene) over time (*p* > 0.05). A main effect for time was detected for α-tocopherol and further analysis suggests an increase at 2 and 4 h versus baseline (*p* < 0.05, Fig. 4). In addition, analysis of lycopene showed a time × group interaction effect (*p* < 0.05). Lycopene decreased 2 h post exercise during the high intensity intermittent walking trial compared to baseline and post-exercise (*p* < 0.05) (Fig. 5).

## Discussion

This study is the first to examine the transient effects of HIIW on biomarkers of oxidative stress and inflammation over time. While no significant changes were observed, IL-6, TNF-α and endothelin-1, all increased post exercise irrespective of exercise intensity or pattern. Such changes were accompanied by an antioxidant response, whereby α-tocopherol rose (HIIW and CMW data) and lycopene decreased (HIIW; pre- and post-exercise versus 2 h).

A main finding of this study is a bout of walking, regardless of intensity or pattern, promoted a systemic increase in IL-6 and TNF-α. Both cytokines peaked immediately post exercise, remained elevated at 4 h and decreased thereafter. These results complement other findings that have shown a rise in plasma IL-6 following both moderate and vigorous exercise (Harris et al. 2008; Scott et al. 2011). Similarly, Leggate et al. (2010) reported moderate and high intensity intermittent cycling stimulated a comparable increase in IL-6 immediately post exercise, which gradually returned to baseline at 23 h. Normally, post-exercise cytokine kinetics are dictated by exercise intensity, with prolonged strenuous bouts stimulating a greater response, compared to shorter intense bouts (Suzuki 2018). Plasma IL-6, derived from skeletal muscle, usually increases exponentially immediately post exercise, stimulating an anti-inflammatory cascade, giving rise to IL-1ra, IL-10 while blunting TNF-α (Petersen and Pedersen 2005). Thus, a rise in post-exercise IL-6 has evolved into an important modulating agent in immunosuppression, however, this is not a consistent finding in the current study, as TNF-α exhibits different kinetics, increasing post exercise in both trials and remaining elevated in the circulation for longer. This is not the first reports of such a rise, as Scott et al. (2011) reported TNF-α increased following 60 min of moderate and vigorous exercise. Numerous mechanisms may account for this rise in IL-6 including reduced glycogen availability, energy expenditure or changes in calcium and stress hormone secretion (i.e. catecholamines, growth factors or cortisol) (Peake et al. 2007). While the current findings are consistent with exercise-induced muscular IL-6 secretion and subsequent appearance in the vasculature, perhaps the unaccustomed fast treadmill walking simply stimulated a pro-inflammatory response characterised by the secretion of TNF-α (Ostrowski et al. 2001). It has also been hypothesised that blood sampling methods may also stimulate such a reaction (Haack et al. 2002). Dixon and colleagues (2009) compared sampling techniques and reported blood drawn via cannula caused a fourfold increase in pro-inflammatory mediators compared to marginal changes with venepuncture. Much of this response is understood to stem from a local inflammatory response within the indwelling vein and may account for the sustained elevation in TNF-α during the post-exercise period, whereas the exercise-induced IL-6 response gradually normalised (Thompson and Dixon 2009). Typically, TNF-α mediates systemic inflammation and is expressed in response to infection or tissue injury (Cawthorn and Sethi 2008). In many respects, the same cytokines and chemokines are released during the acute phase response but differ in the order of activation, starting with TNF-α (Petersen and Pedersen 2005). Within this pro-inflammatory model, neutrophil chemotaxis upregulates as does the expression of adhesion molecules, selectins and chemokines, exacerbating the pro-inflammatory state (Mihara et al. 2012). Perhaps the inclusion of a resting control group might have provided clarity on the source of cytokine secretion, but this aside, the current study is the first to show a bout of HIIW has such an effect and also highlights the complexities in analysing cytokine networking and inflammatory regulation post exercise.

ROS may also prove a key mediator within the exercise-induced cytokine response through the stimulation of NF-κB, which subsequently regulates inflammation (Kramer and Goodyear 2007). Steinberg et al. (2007) provided data for a possible interaction after reporting an accompanied rise in lipid peroxidation and cytokine secretion. However, the current study showed no such changes in biomarkers of oxidative stress indicating the mechanism with walking may be more complex. It is possible that the exercise intensities increased cytokine concentrations independent of oxidative stress, or perhaps oxidative parameters outside the scope of this study may have contributed. Moreover, the activation of mitogen-activated protein kinases changes in calcium homeostasis, or impaired glucose availability may also attribute to the cytokine response (Fischer 2006). As participants undertook exercise while fasting the latter might be possible, coordinated via AMP-activated protein kinase signalling (Li and Gleeson 2005).

HIIW did not promote an increase in ROS or other related biomarkers. Several studies reported increased free radical production and oxidative damage with vigorous to maximal bouts of exercise (Alessio et al. 2000; Quindry et al. 2003; Davison et al. 2012). It has also been reported that as little as 5 min of exercise at 70% $\dot{V}{\text{O}}_{2} \max$ can stimulate oxidative damage (Fogarty et al. 2011). Thus, the exercise intensity, duration and mode appear to be key mediating factors for the production of oxidative stress (Parker et al. 2014). Whilst humans are equipped with a sophisticated network of antioxidant defence mechanisms designed to neutralise ROS, an intense or extended bout can lead to lipid, protein and/or DNA damage (Valko et al. 2007; Powers and Jackson 2008). Perhaps the exercise trials employed did not sufficiently overwhelm antioxidant capacity, preventing a rise in oxidative stress and protection against molecular damage. Alternatively, several studies have reported greater antioxidant status in trained participants (Powers and Jackson 2008; Djordjevic et al. 2012), indicating that current participants may possess efficient antioxidant capability, allowing them to manage ROS more effectively, given the enzymatic antioxidant response associated with exercise training. The observed reduction in lycopene following HIIW provides the best example of this antioxidant action. Lycopene has the capacity to neutralise potent-free radicals (hydroxyl, hypochlorous acid and peroxyl), offering protection against lipid peroxidation and lipoprotein modification while stimulating enzymatic antioxidants (Pennathur et al. 2010). Scavenging peroxyl radicals may preserve α-tocopherol, a prominent chain-breaking antioxidant, possibly translating to the main effect for time at 2 and 4 h (Stahl and Sies 2003; Powers and Jackson 2008). Davison et al. (2012) demonstrated the importance of α-tocopherol, as a reduction prompted a rise in lipid peroxidation and free radical production. Interestingly, IL-6 is known to upregulate enzymatic antioxidant expression, so it may conceivably mobilise non-enzymatic antioxidants, given its proposed role in adipose tissue and adipokine/myokine crosstalk (Vassilakopoulos et al. 2003; Trayhurn et al. 2011). Overall, the lack of change in oxidative parameters, some of which are potent signalling molecules (i.e. H_2_O_2_), could mean the acute activation of some redox-sensitive adaptations are missed (Veal et al. 2007). Perhaps the exercise-induced increase in cytokine activity was triggered by minimal, subtle changes in ROS, or by other redox variables, not directly evident in the current study. Further, the precise post-exercise sampling time points may account for the negligible change, as oxidative parameters are variable in nature and respond to stimuli differently over time (Michailidis et al. 2007). Alternatively, the results could potentially suggest another mechanism of action, at least in this exercise model, though at this time it is difficult to conclude and further research, possibly including analyses of muscle tissue, may be necessary to provide more mechanistic insight.

Finally, a main effect for time was detected for endothelin-1. Endothelin-1 participates in vasoconstriction, free radical production, proliferation and platelet activation (Böhm and Pernow 2007). Therefore, determining its acute function is desirable as previous research has mainly focused on aerobic exercise training (Maeda et al. 2009). Within the acute exercise model, McClean et al. (2015) also reported endothelin-1 increased immediately following 30 min of aerobic exercise at 55% $\dot{V}{\text{O}}_{2} \max$. The rationale for the increase may be associated with other vasoactive or pro-inflammatory mediators or exercise-induced changes in catecholamines but further research is necessary to explore this pathway. The findings suggest that the increase in endothelin-1 and its associated detrimental effects are overridden as improvements in vascular function persist.

This randomised control trial is not without limitations. First, including a resting control group within the experimental design would add clarification to the inflammatory response, attributing changes to the sampling method or indeed the bouts of exercise. IL-6 is a responsive cytokine but our analysis and subsequent interpretation is based on its perceived post-exercise anti-inflammatory actions, as such this could be considered a minor limitation. Furthermore, analysing a more diverse array of cytokines (namely IL-1ra and IL-10) during the post-exercise period would allow a comprehensive investigation of the systemic and delayed inflammatory response. Second, stringent dietary control and analyses might provide a commentary on glycogen availability as well as antioxidant status, given both interfere in signalling processes that regulate cytokine secretion. Likewise, sleep quality has recently emerged as potential influence over markers of immunity and as such should be considered carefully in the design of studies addressing cytokine activity and exercise. Future investigations should place an emphasis on clinical or perhaps elderly populations, given global improvements in life expectancy and the influence of inflammation during ageing. Finally, further trials should attempt to clarify the intracellular mechanisms relating to oxidative stress and cytokine activity within the exercise model.

In summary, the results indicate that walking modulates systemic cytokine secretion independent of oxidative stress. Walking also appears to promote an antioxidant response and collectively improves vascular function. Theoretically, the findings (i.e. no major changes in inflammatory or oxidative parameters) suggest HIIW and CMW are equally effective and stimulate similar physiological responses. Given time commitments are often cited for inactivity, HIIW may provide an alternative method for engaging in daily physical exertion. Further research is necessary to establish the precise mechanisms of action as well as determining the longitudinal efficacy of intermittent bouts of walking within the broader spectrum of public health.

## Abbreviations

ANOVA: Analysis of variance
Asc·: Ascorbyl radical
CMW: Continuous moderate walking
DMSO: Dimethyl sulfoxide
DNA: Deoxyribonucleic acid
EDTA: Ethylenediaminetetraacetic acid
ELISA: Enzyme-linked immunosorbent assay
EPR: Electron paramagnetic resonance
H_2_O_2_: Hydrogen peroxide
HIIW: High intensity intermittent walking
HPLC: High performance liquid chromatography
IL-: Interleukin
LOOH: Lipid hydroperoxides
MAPK: Mitogen-activated protein kinase
NF-κB: Nuclear factor kappa B
P: Statistical significance
ROS: Reactive oxygen species
RPM: Revolutions per minute
SEM: Standard error of the mean
TNF-α: Tumour necrosis factor alpha
$\dot{V}{\text{O}}_{2} \max$: Maximal oxygen uptake
